# How does COVID‐19 impact treatment decision‐making for clinicians in localised kidney cancer

**DOI:** 10.1002/bco2.57

**Published:** 2020-11-23

**Authors:** Katharina Beyer, Mieke Van Hemelrijck, Netty Kinsella, Ravi Barod

**Affiliations:** ^1^ Translational Oncology and Urology Research (TOUR) King’s College London London UK; ^2^ Department of Urology Royal Marsden Hospital London UK; ^3^ Specialist Centre for Kidney Cancer Royal Free Hospital London UK

Kidney cancer (KC) is the seven most common cancer in the United Kingdom; about 56% of new cases classified as localized disease. Although there are several treatment options which achieve similar oncological outcomes for those with localized disease, they all present different side effect profiles,^1^ making appropriate treatment selection paramount to optimize quality of life.

The COVID‐19 pandemic has complicated the process of decision making. Official guidance called for non‐urgent cancer care to be rationed, delayed and/or adapted. Hence, the British Association of Urological Surgeons (BAUS) recommended that patients diagnosed with localized KC were offered a period of surveillance rather than curative treatment.^2^

The impact of COVID‐19 medical guidance on patients was described by kidney patient associations in the United States and United Kingdom (KCCURE and KCUK) in small snapshot surveys, exploring patient experience, anxiety, and their management.^3^, ^4^ They found that anxiety was high in respect to both cancer and COVID‐19 and its implications for treatment and follow‐up.

Building on these initial observations, we conducted a cross‐sectional, web‐based survey amongst health care professionals (HCPs) delivering localized KC treatments, to understand the barriers and facilitators to supporting patients in their treatment decisions. Our multidisciplinary research team followed the Checklist for Reporting Results of Internet E‐Surveys (CHERRIES). The ethics board of King's College London approved the survey as a Minimal Risk Study.

The survey (28 questions) was distributed via Twitter on May 16th, 2020 for 22 days using SmartSurvey. Fifty‐eight respondents (36 from the United Kingdom and 22 from outside of the United Kingdom) completed the survey, of which 43% were United Kingdom doctors, 19% were United Kingdom nurses and 38% non‐UK doctors. 31% of the UK participants were working at Specialist Centres tertiary referral hospitals, 47% at District General or Teaching hospitals and 22% only indicated NHS Trust.

Five main themes emerged from the survey: diagnostics, treatment, consultations and supportive care, HCP satisfaction, and delivery of future KC care. Due to disparities in healthcare guidelines followed by each country, we have focused our report on the United Kingdom.

In the context of diagnostics, Oderda et al noted that the delays observed during the pandemic have severely impacted on patients through lengthening of both diagnostic and treatment waiting lists. They emphasized the need for healthcare authorities to develop strategies to catch up with diagnostics.^5^ This was confirmed in our survey as 75% of survey respondents highlighted disruption to the diagnostic pathway in the United Kingdom, compared to only 27% for non‐UK respondents: reduced access to imaging (69%), reduction or no access to kidney biopsy (78%), delivering diagnostic consultations via phone (83%) and video call (25%). Moreover, the discussion of those patients newly diagnosed in the Multidisciplinary Team meeting (MDT) posed another problem. During COVID‐19 these were moved to virtual platforms overnight. The MDTs are a forum for discussion, knowledge transfer, and learning. When asked whether these should remain as virtual events, 55% of nurses in the UK felt that these should revert to face‐to‐face meetings, compared to only 28% of UK doctors. The opportunity to connect with the wider team is a unique experience with virtual MDTs possibly leading to a loss of professional understanding and social interaction.

With respect to treatment during Covid‐19, the EAU Guidelines Office Rapid Response Group is recommending postponement of surgery by 6 months where progression is unlikely.^6^ Fifty‐eight percent of UK respondents reported that deferring treatment for 0‐12 months for T1a disease or deferring treatment for 3‐6 months in T1b disease (53% of respondents) or a delay of 0‐6 month for T2 lesions (83% of respondents) would adversely affect the oncological outcomes. This might also in part explain the high level of dissatisfaction in respect to available treatment options, where none of the UK nurses who responded and only 28% of UK doctors were satisfied with the treatment options.

For consultations and supportive care, the main patient focus has been to prevent unnecessary risk of exposure to COVID‐19. To this end, telephone and video consultations were implemented across the United Kingdom in line with BAUS guidance. Boehm et al assessed the willingness of patients to telemedicine (video consultations) and their results suggested that 54% were willing to undertake telemedicine consultations.^7^ However, this survey identified that the majority of treatment consultations in the United Kingdom were carried out via telephone during COVID‐19 (86% for treatment consultation) instead of video consultations (22%). It is, therefore, not possible to understand if HCPs would have felt more satisfied and prepared to provide supportive care using telemedicine consultations.

The UK’s deferred treatment plan was reported by the respondents as a contributor to HCP dissatisfaction. Only 36% of UK respondents were able to perform partial nephrectomies and 8% ablation. As reported by KCCURE’s and KCUK’s snapshot surveys, patients reported experiencing a high level of anxiety during the COVID‐19 period. Therefore, it is not surprising that 47% of UK respondents confirmed an increase in the number of patients contacting their service. This, combined with widespread re‐deployment of health care professionals (56% of the medical team and 81% of the nursing team) may explain the reason why only 47% of UK nurses and doctors felt satisfied to very satisfied with the service they provided during the peak of the pandemic. With a demonstrable increase in cancer patient anxiety,^3^, ^4^ consideration should, hence, be given to the merits of staff redeployment versus patient safety if a second wave pandemic was realized.

For the delivery of future KC care, 78% of UK respondents agreed there is a need for additional imaging (78%), theatre (100%), inpatient (78%), outpatient capacity (69%) and manpower resources (58%). Responding to the additional needs will be challenging, but necessary.^5^ This includes a stratified approach to patient assessment in relation to anxiety and depression, as a result of the increase in waiting times. Moreover, both doctors and nurses reported a high level of moral distress. 67% of respondents felt distressed to worst possible distress. This should be taken seriously by policy makers and hospital executives going forward.

A limitation was the distribution of the survey via twitter, however the CHERRIES statement, which aims to reduce selection bias, guided our understanding of the sample (self)selection (see online Appendix).^8^

Our survey has shown high levels of dissatisfaction among HCPs regarding the standard of care delivered during the COVID‐19 pandemic in the United Kingdom, suggesting that there is a need to re‐visit the guidelines. It is important to ensure the diagnostic pathway is not disrupted, ensure the ability to use video consultations is available, prevent the medical team and particularly CNSs to be redeployed and all available treatment options such as the ability to perform surgery in COVID‐19 cold sites are assured. Our findings should be used to inform policy on KC care provision in the event of a second wave or a future healthcare crisis.

## CONFLICT OF INTEREST

Grants were received from Kidney Cancer UK and the Royal Marsden Charity.

## Supporting information

Table S1
